# Plant-Based Diets in the Reduction of Body Fat: Physiological Effects and Biochemical Insights

**DOI:** 10.3390/nu11112712

**Published:** 2019-11-08

**Authors:** Rami S. Najjar, Rafaela G. Feresin

**Affiliations:** Department of Nutrition, Georgia State University, Atlanta, GA 30302, USA

**Keywords:** diet, nutrition, vegetarian, vegan, microbiome, TMAO, PPAR, weight loss, obesity

## Abstract

Obesity affects over one-third of Americans and increases the risk of cardiovascular disease and type II diabetes. Interventional trials have consistently demonstrated that consumption of plant-based diets reduces body fat in overweight and obese subjects, even when controlling for energy intake. Nonetheless, the mechanisms underlying this effect have not been well-defined. This review discusses six major dietary mechanisms that may lead to reduced body fat. These include (1) reduced caloric density, (2) improved gut microbiota symbiosis, (3) increased insulin sensitivity, (4) reduced trimethylamine-N-oxide (TMAO), (5) activation of peroxisome proliferator-activated receptors (PPARs), and (6) over-expression of mitochondrial uncoupling proteins. Collectively, these factors improve satiety and increase energy expenditure leading to reduced body weight.

## 1. Introduction

The Centers for Disease Control and Prevention estimates that 35.6% of adults age ≥20 years are obese, and more than 1.4 billion adults are overweight worldwide [1,2]. Obesity may shorten human lifespan by 4–7 years, presumably due to the associated increased chronic disease risks for type II diabetes mellitus (T2DM), cardiovascular disease (CVD), and cancer [3,4,5]. While a variety of environmental factors influence the development of obesity, diet has a significant influence on adiposity [6].

Plant-based diets have been consistently associated with reduced body weight in a multitude of interventional trials [7,8]. In a comparative, randomized study, obese, middle-aged subjects (*n =* 62) were assigned to consume ad libitum either an omnivorous, semivegetarian, pesco-vegetarian, vegetarian, or vegan diet for six months [9]. The greatest weight loss after 6 months was in the vegan (−7.5% of body weight) and vegetarian (−6.3% of body weight) subjects, compared with the other groups (about −3.2% of body weight). In a large, prospective clinical trial with overweight and obese subjects diagnosed with T2DM, subjects were randomly assigned to either a low-fat vegan diet (*n* = 68) or a control, habitual diet (*n* = 45) with ad libitum intake [10]. After 22 weeks, subjects consuming the vegan diet lost 5.1 kg of body weight compared to control (+0.1 kg). In a 12-month randomized controlled trial, overweight and obese subjects with T2DM or CVD pathology were assigned to either consume an ad libitum whole-foods, plant-based diet (*n* = 33) or receive standard medical care (n = 32) [11]. Subjects consuming the plant-based diet lost 11.5 kg (*p* < 0.0001) compared with the control group, which did not significantly change weight (−1.6 kg, *p =* 0.13). In the Adventist Health Study-2, nonvegetarians, semivegetarians, pesco-vegetarians, lacto-ovo vegetarians, and vegans (*n =* 60,903) were found to have significantly different body mass indexes (BMIs) [12]. A stepwise, linear decrease in BMI was observed in accordance with a stepwise reduction in animal product consumption, from nonvegetarians with the highest average BMI (28.8 kg/m^2^) to vegans with the lowest average BMI (23.6 kg/m^2^). On average, vegans were the only dietary group in this cohort to be considered normal weight. T2DM incidence was also reduced in a stepwise fashion, with the highest rates in nonvegetarians compared to vegans. 

**Figure 1:**
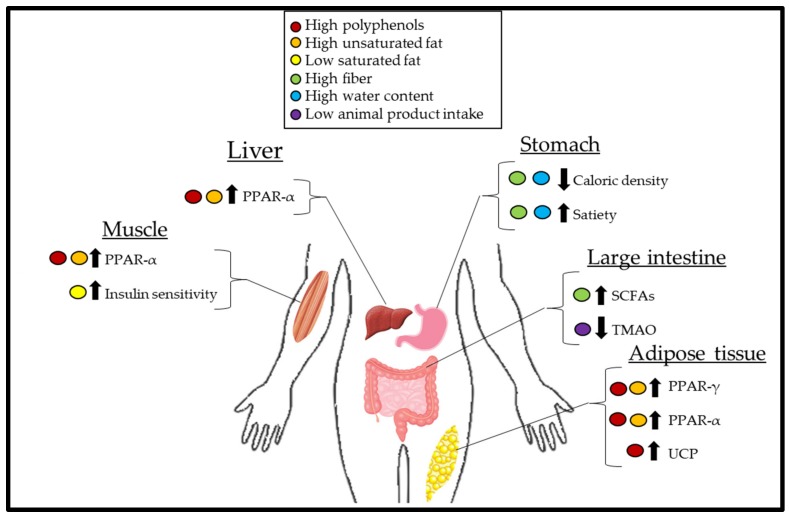
Physiological effects of a plant-based diet and the interplay of organ systems in the context of weight loss. Polyphenols and unsaturated fatty acids can act on muscle, liver, and adipose tissue to upregulate the expression of peroxisome proliferator-activated receptor (PPAR)-α, which increases β-oxidation leading to a reduced circulating pool of free fatty acids (FFAs), thus decreasing the availability of FFA for adipose tissue uptake and hypertrophy. Additionally, polyphenols and unsaturated fatty acids can act on adipose tissue to increase the expression of PPAR-γ, which results in FFA uptake by adipose tissue, further decreasing the FFA pool. A decreased FFA pool improves insulin sensitivity leading to increased thermogenesis due to improved glucose handling. A decreased consumption of saturated fats, which are primarily derived from animal-based foods, further improves insulin sensitivity. Polyphenols also act on uncoupling proteins (UCPs) within the mitochondria, increasing thermogenesis. Foods of a lower caloric density, due to being higher in fiber and water, often take up more stomach volume than calorie-dense foods, leading to overall reduced caloric intake and early satiety. Increased short-chain fatty acid (SCFA) synthesis from fiber fermentation due to microbes in the gut increases satiety hormones and delays gastric emptying. Favorable gut microbes resulting from decreased animal product consumption decreases trimethylamine-N-oxide (TMAO) synthesis. Decreased TMAO increases the presentation of more metabolically active beige adipose tissue.

Despite the consistency by which plant-based diets are associated with reduced body weight [7,8], the mechanisms by which this occurs have not been well-defined. The objective of this review is to discuss the potential physiological and biochemical mechanisms that contribute to the reduction in body fat in overweight or obese subjects consuming plant-based diets. Cumulatively, plant-based diets may reduce body fat because of the overall decreased caloric intake and increased energy expenditure due to increased thermogenesis (Figure 1).

## 2. Mechanisms of Weight Loss

### 2.1. Calorie Density 

**Figure 2:**
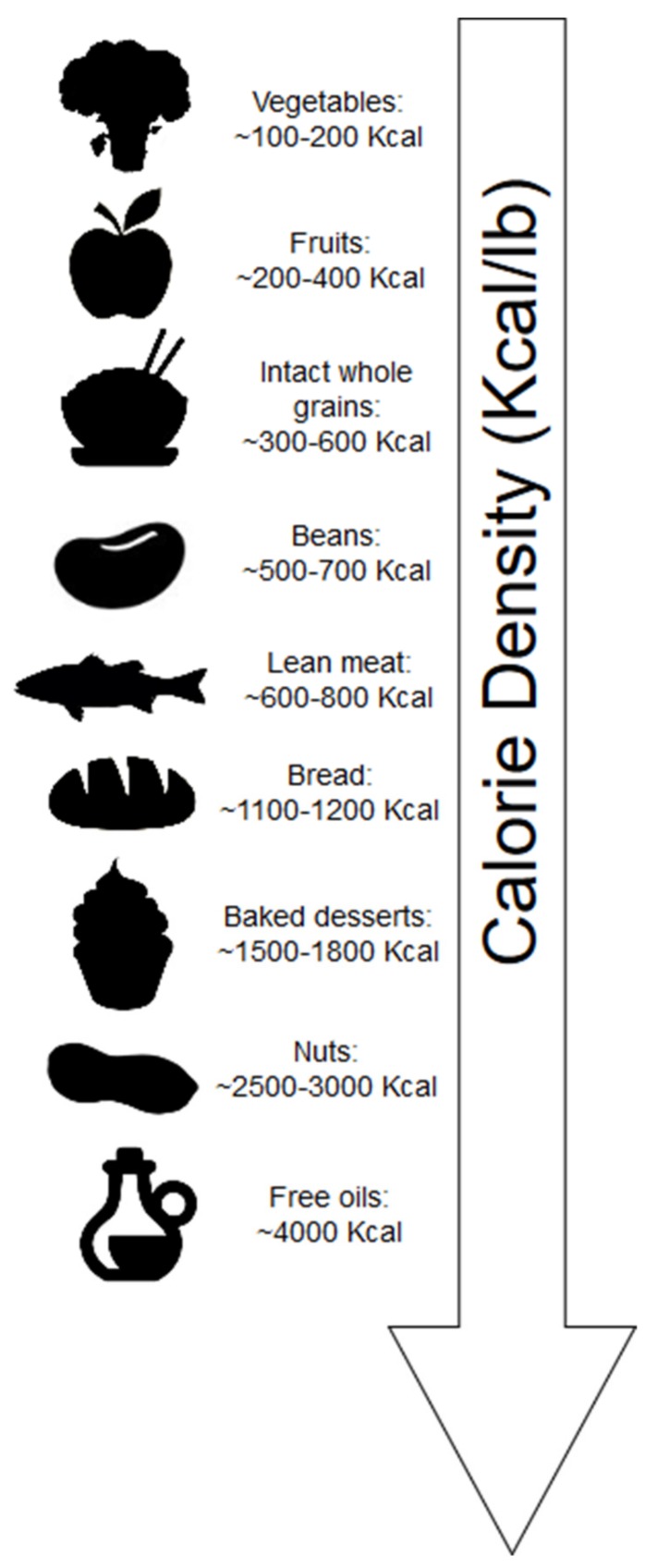
Average calorie density of foods in kcal/lb. Foods higher in water and fiber are typically more calorie dilute. Dry foods, foods with less fiber, and/or foods that are higher in fat content are more calorie dense.

Calorie density refers to the number of kilocalories (kcal) per unit weight of food. Whole plant-foods contain mostly water by weight; thus, these foods generally have a low-calorie density (Figure 2). Additionally, fiber constitutes weight yet does not contribute fully to the expected kilocalories of digestible carbohydrates. Short-chain fatty acid (SCFA) produced by bacteria of the gut due to fiber fermentation contributes ~2 kcal/g [13].

Individuals generally consume the same weight of food during meals, as such, the clear advantage of consuming foods low in calorie density is that these foods can contribute to stomach volume, feelings of fullness and satiety while maintaining low caloric intake [14]. Minimally processed foods of plant origin, which are both high in water content and fiber, are generally lower in calorie density (Figure 1). Exceptions arise for foods with minimal water, such as bread (including whole grain), which is dry; thus, the calorie density is increased in these foods. Additionally, nuts, which are both dry and contain calories derived mostly from fat, have significantly greater calorie density.

Despite the potential of these dry plant-foods to contain significant fiber content, factors affecting dryness may be more influential in mitigating energy intake. For example, when obese and overweight women (age 35–70 years) were randomly assigned to consume either three apples (*n* = 16), three pears (*n =* 16), or three oat cookies (*n =* 17) every day for 10 weeks as part of an ad libitum diet, those who consumed the apples and pears reduced their calorie intake by ~25 and ~20 kcal/d, respectively [15]. These calorie reductions were associated with significantly reduced body weight in both apple (β= −0.92 kg, *p =* 0.0001) and pear groups (β= −0.84 kg, *p =* 0.0004). However, despite the oat cookies containing a similar fiber content (~6 g), those in the oat cookie group did not significantly alter their energy intake and body weight. Considering that the weight of foods consumed largely impact caloric intake [14], the water content of the fruits likely contributed substantially to these effects, since three apples and three pears are both 300 g, whereas the three oat cookies were 60 g. It should be noted that the food matrix is also of significance, and the disrupted fiber of the oats may have reduced satiety. For example, a randomized, crossover study by Flood-Obbagy and Rolls [16] indicated that consumption of whole apple segments with undisrupted fiber (intact) but not apple juice, with added fiber, before an ad libitum meal, resulted in reduced energy intake.

Consuming foods of lower calorie density may be more advantageous for sustainable weight loss compared to reducing portion sizes. In a crossover study, during ad libitum intake under isocaloric conditions, young (19–35 years), normal weight (22.6 kg/m^2^) women (*n =* 24) subjects with 25% reduced food portions consumed 10% fewer calories than standard condition meals (100% portion and 100% energy density) [17]. However, when energy density was reduced by 25%, while keeping food weight constant, subjects consumed 24% fewer calories, and satiety was improved compared to the standard condition meal. In general, reducing the energy density of the overall diet is a suitable strategy for weight loss [18].

In the context of a plant-based diet, in a single-arm investigation, obese men and women (age 25–64 years, *n* = 19) who consumed a traditional Hawaiian diet ad libitum for 3 weeks consisting of mostly starchy tubers, fruits, and vegetables, with restricted intake of chicken and fish (142–198 g/d), reduced their average daily energy intake from 2594 to 1569 kcal [19]. Carbohydrates, which are mostly derived from plants, increased from 51% to 78% of total energy, and subjects subsequently lost ~17 lb. In another single-arm intervention, ad libitum intake of a raw, plant-based diet for 4 weeks containing 11.8 servings/d of fruits and 16 servings/d of vegetables resulted in a daily ~700 kcal deficit with a weight loss of ~14.75 lb [20,21].

### 2.2. Role of the Gut Microbiota

The gut microbiome can influence energy balance and is a major site of small molecule production, which can influence satiety and gut inflammation [22]. In general, the Bacteroidetes taxa have been associated with reduced adiposity compared with Firmicutes, which are associated with obesity [23]. In a randomized, crossover trial, a 20% increase in Firmicutes was associated with ~150 greater kcals absorbed, whereas a 20% increase in Bacteroidetes was associated with ~150 fewer kcal absorbed in lean subjects [24]. Indeed, weight loss through a mere reduction in total kcal can shift bacterial gut populations in obese individuals from Firmicutes to Bacteroidetes [23]. Highlighting this effect, lean subjects (*n =* 12) who were overfed (3400 kcal/d) lost fewer calories in their stools (indicating greater energy absorption) compared to a 2400 kcal/d diet [24]. Thus, simply consuming more kcal in a day can shift the gut microbiome to more obesogenic taxa. Despite these compelling findings, associations between specific bacterial taxa and health status have not been fully characterized [25] and require further investigation.

Highlighting the complexity of the gut microbiota, microbiome dysbiosis associated with obesity [26] promotes bile acid fermentation. More specifically, a small number of anaerobic bacteria promote deconjugation and dehydroxylation of primary bile acids resulting in the presence of secondary bile acids. These secondary bile acids have increased hydrophobicity and pKa, thereby increasing their absorption into the gut wall [27]. Likely as a compensatory mechanism, secondary bile acids can bind to nuclear farnesoid X receptor and G-protein-coupled bile acid receptor 1 of various tissues, increasing energy expenditure and β-oxidation [28]. However, these secondary metabolites may also decrease beneficial bacteria populations, such as *Lactobacillus* [29]. In 3T3-L1 preadipocytes cultured with heat-killed *Lactobacillus plantarum* K21, significantly fewer lipid droplets accumulated during differentiation compared to control [30]. Additionally, mice fed a high-fat diet supplemented with a *Lactobacillus plantarum* K12 probiotic had significantly decreased body weight compared to high-fat diet fed control [30]. The probiotic-supplemented mice also had a decreased food efficiency ratio, suggesting less energy absorbed from the food consumed, decreased leptin, and decreased triglycerides compared to high-fat diet control. 

In addition, decreased body weight facilitated by appetite suppression in those consuming plant-based diets is largely mediated by the gut microbiome. This effect has been characterized as the “second meal effect”, described as the phenomenon by which the first meal consumed at an earlier time suppresses one’s appetite during a later meal, leading to reduced caloric intake and improved glycemic control [31,32,33,34,35]. In a randomized, crossover trial with healthy young men (*n =* 43), an ad libitum, macronutrient matched (19% protein, 53% carbohydrate, 28% fat) high-protein legume-based meal (derived primarily from fava beans and split peas) led to increased fullness and increased satiety compared to a high-protein animal-based meal (derived primarily from veal) [36]. Furthermore, 95 kcal less of the legume-based meal was consumed compared to the animal-based meal. This process is largely dependent on the colonic fermentation of indigestible fibers found in whole grains and starchy legumes producing butyrate, propionate, and acetate SCFAs [37]. Besides being a direct source of energy for colonocytes, SCFAs act as substrates for G-protein coupled receptors on various tissues, stimulating the release of peptide YY (PYY), a hormone that reduces appetite and food intake, as well as glucagon-like peptide (GLP-1), a hormone that delays gastric emptying [38].

In a randomized crossover trial, 16 healthy subjects, both men and women (mean age 23.8 years), consumed brown beans during an evening meal, which resulted in increased breath SCFAs, increased PYY, and decreased ghrelin, a hormone that stimulates appetite and fat storage, at breakfast compared to a white bread meal [39]. Furthermore, blood glucose and insulin secretions during the breakfast were also significantly decreased. In hyperinsulinemic subjects randomized to high-wheat fiber consumption (*n =* 14) or low-wheat fiber consumption (*n =* 14), increased SCFAs and GLP-1 were observed only in the high-wheat fiber group; however, these microbial adaptations took 1 year to develop [40]. Gut bacteria populations, which digest these fibers in wheat and beans and produce SCFAs, are found in much smaller quantities in those consuming animal-based diets [41]. In 10 healthy subjects (age 21–33 years) who participated in a nonrandomized, controlled feeding study [41], it was observed that during the plant-based feeding, acetate and butyrate were significantly greater than when subjects consumed the animal-based diet. Furthermore, bile salt hydrolase activity and secondary bile salts significantly increased during animal-based feeding, suggesting the suppression of beneficial bacterial populations [29]. Further illustrating these effects, in a single-arm intervention, six obese subjects (women *n =* 5, men *n =* 1) who were diabetic and/or hypertensive decreased their gut Firmicute population and increased Bacteroidetes by consuming a strict vegan diet for 4 weeks [42]. In general, cross-sectional and interventional data suggest a higher proportion of beneficial SCFA-producing bacteria in those consuming vegan versus omnivorous diets [43].

### 2.3. Insulin Sensitivity, Carbohydrates, and Diet-Induced Thermogenesis

Obesity is tightly linked with the development of insulin resistance, the underlying cause of T2DM [44]. While current nutrition recommendations to manage T2DM largely revolve around the management of carbohydrates [45], it is misleading to assume that insulin resistance is caused by excessive carbohydrate intake. Certainly, refined carbohydrate sources and added sugars are associated with insulin resistance and T2DM [46,47]. However, the consumption of refined carbohydrates and added sugars may merely associate with unhealthy lifestyle habits and weight gain, which increases the risk for these disease states [48]. Della Pepa et al. [49] recently compiled evidence from epidemiological and interventional studies showing that consumption of whole grains, a rich carbohydrate source, reduces the risk for T2DM. In addition, increasing carbohydrate consumption from unrefined sources can improve insulin sensitivity. In a single-arm investigation of subjects (*n* = 20) with T2DM, switching to a weight-maintaining, high-fiber, plant-based diet (70% carbohydrate by composition) nearly eliminated the need for exogenous insulin injections within 2 weeks [50].

A large body of evidence supports the hypothesis that insulin resistance is a pathology characterized by lipotoxicity [51]. Saturated fat (SFA), particularly palmitate, can inhibit insulin signaling in myocytes at the cytosolic level due to the accumulation of free fatty acid (FFA) intermediates, ceramide and diacylglycerol [52]. Additionally, excessive palmitate oxidation facilitates mitochondrial dysfunction, which reduces ATP synthesis, thus lowering ATP bioavailability for insulin signaling and increasing oxidative stress [53,54]. Plant-based diets may contain low levels of SFAs, which are mainly derived from oils such as palm and coconut oil; however, SFAs in the American diet are primarily derived from animal-based foods (Table 1). This may explain why plant-based dietary patterns are associated with reduced insulin resistance compared to animal-based diets [55].

Modulating FFA concentrations either pharmacologically or via lipid infusion reveals that increased FFA reduces insulin sensitivity in humans [57,58,59,60]. It should be noted that FFAs can be elevated by dietary means (consumption of SFA-rich foods) or endogenously in the case of obesity [52,61,62]. This largely explains why those who are obese that undergo significant weight loss, either with calorie restriction or bariatric surgery, can reverse insulin resistance [63,64,65].

Increased insulin sensitivity in itself may directly impact body weight. Indeed, subjects who are insulin resistant have reduced energy expenditure from carbohydrate ingestion due to impaired glucose handling [66]. Since carbohydrate and whole grain consumption can independently increase energy expenditure [67], increasing insulin sensitivity may significantly impact body weight. This may explain the observation as to why a variety of interventions have found similar caloric intakes between nonvegetarian and plant-based subjects, yet greater reductions in fat mass within the plant-based arm [68,69,70,71]. A 2005 randomized, ad libitum investigation by Barnard et al. [72] found that overweight women (*n =* 29) who consumed a plant-based diet for 14 weeks lost 2 kg more than the control group (*n =* 30) despite near equivalent caloric intakes. However, regression analysis revealed that the observed increase in the thermic effect of food significantly contributed to the weight loss that occurred in those consuming the plant-based diet.

Compared to a calorie-restricted American Diabetes Association (ADA) diet, subjects randomly assigned to consume an ad libitum vegan diet (*n =* 49) for 74 weeks significantly reduced glycated hemoglobin (a crude measure of insulin resistance) compared to the ADA dietary group (*n =* 50) in those who did not alter their medications (−0.40% in vegan subjects vs. +0.01% in ADA subjects) [68]. Interestingly, both dietary groups had similar caloric intake (1366 kcal in the vegan group vs. 1422 kcal in the control group, *p =* 0.90). While weight was not significantly different between groups (*p* = 0.25), waist circumference trended towards significance in subjects within the vegan group compared to the control group (−4.2 cm and −1.8 cm, respectively, *p =* 0.06) suggesting reduced fat mass [73].

In a 16-week randomized trial, an ad libitum vegetarian diet trended towards promoting greater reductions in intramyocellular lipids of the thigh (*n =* 38) compared to the hypocaloric ADA diet in diabetic individuals (*n =* 37) [70]. Additionally, weight loss was nearly twice as great in the vegetarian group than the hypocaloric diet group (−6.2 kg vs. −3.2 kg, respectively). In a similarly designed investigation, subjects consuming a vegan diet (*n =* 38) experienced improved β-cell function and increased insulin sensitivity versus the control group (*n =* 37) [71]. This occurred despite nearly equivalent caloric intakes (1582 kcal for control group vs. 1450 kcal for vegan group, *p =* 0.69). However, body weight, particularly fat mass, was significantly reduced in the vegan group compared to the control group (39.1 to 39.5 kg and 42.0 to 38.1 kg, respectively, *p* < 0.001). It is likely that the improved insulin sensitivity observed in these trials contributed to reduced adiposity by increasing energy expenditure associated with glucose handling.

### 2.4. Obesogenic Effects of Trimethylamine-N-Oxide

L-carnitine can be found in small amounts in plant-based foods such as avocado and beans; however, red meats and other animal products are the main sources of L-carnitine [74]. On the other hand, while red meat and eggs are rich sources of choline, plant-based foods such as soybeans, potatoes, and most beans are also considered good sources of choline [56]. Choline and L-carnitine are metabolized by gut bacteria to produce trimethylamine (TMA) [75]. In the liver, TMA is a substrate for flavin-monooxygenase-3 (FMO3) and is oxidized to form trimethylamine-N-oxide (TMAO). TMAO is tightly associated with the development and risk of atherosclerosis by potentially inhibiting reverse cholesterol transport and promoting thrombosis [76,77].

In a cross-sectional investigation (*n =* 137), subjects with the highest concentrations of TMAO had the greatest BMI and waist circumference [78]. However, these findings were confounded by greater energy intake within the highest TMAO bracket. Interestingly, when correlation analysis was conducted adjusting for BMI and energy intake, those with the highest TMAO intake had the greatest degree of insulin resistance and adipose tissue dysfunction, which may inherently result in a decreased thermic effect of food. Indeed, a linear association between TMAO concentrations and T2DM incidence has been observed [79]. Additionally, FMO3 knockout mice were protected against high-fat diet-induced obesity. In this murine model, TMAO may mediate this effect by preventing white adipose tissue from becoming more metabolically active and energy intensive as beige adipose tissue. These effects may be clinically relevant as beige adipose tissue is present in both mice and humans [80], although human trials are needed to confirm the effects of TMAO on adipose tissue.

Interestingly, TMAO production does not occur in those consuming vegan diets. For example, a dietary challenge of 250 mg d3-carnitine and an 8 oz steak resulted in a significant increase in plasma TMAO concentrations in an omnivorous subject in a time-dependent manner; however, there was no increase in TMAO concentrations in the vegan counterpart [77]. This effect was replicated in vegan/vegetarian (*n =* 5) and omnivorous subjects (*n =* 5) who consumed 250 mg d3-carnitine alone. Furthermore, fasting vegans/vegetarians (*n =* 23) had significantly less plasma TMAO compared with fasting omnivorous subjects (*n =* 51). This can be attributed to the dominant bacterial enterotype, primarily Bacteroidetes, which does not produce TMA, that resided in these individuals consuming plant-based diets [77]. Further evidence supports this diet-enterotype–TMAO connection, as vegetarians were found to have significantly fewer TMAO-producing bacterial populations than omnivorous counterparts [81]. While the strength of association between TMAO and CVD is strong, more evidence is needed to confirm the association between TMAO and obesity [82].

### 2.5. Unsaturated Fatty Acids and the Role of PPAR

Despite the relatively high caloric density of nuts (Figure 2), nuts surprisingly are not associated with weight gain and, in fact, are associated with reduced body weight and waist circumference [83]. Although a good source of fiber, the mechanisms by which nuts reduce body weight appear to be independent of this nutrient. The mechanisms attributed to nuts and weight loss primarily are due to incomplete mastication of the cell walls, improved satiety, and thermogenic effects [84]. Nuts are relatively low in SFA, and increased thermogenesis may stem from the higher unsaturated fat content of nuts (Table 1), as SFAs (found primarily in dairy and other animal-based foods) may be more obesogenic [85]. High-fat feeding studies illustrate this effect. In a nonrandomized crossover study using radio-labeled carbon, 13C-oleate was oxidized at a 21% greater rate than 13C-palmitate in 10 healthy men [86]. In a 4-week crossover study, 8 obese or overweight subjects consumed high-fat diets (40% of energy, fixed for macronutrient composition) ad libitum [87]. Subjects consumed either a high-SFA diet (24.4% SFA, 12.5% monounsaturated fat (MUFA)) or a high MUFA-rich diet (11% SFA, 22.3% MUFA). Both diets were designed to exceed calorie needs (~3000 kcal/d). Despite nonsignificant differences in kcal consumed on both diets (3003 kcal on SFA-rich diet vs. 2843 kcal on MUFA-rich diet, *p =* 0.16), SFA consumption nonsignificantly increased body weight (+0.6 kg) and body fat percent (+0.8%), whereas MUFA consumption significantly decreased body weight (−1.6 kg) and body fat percent (−1.1%).

A possible explanation is the regulation of peroxisome proliferator-activated receptor (PPAR) by these fatty acids. PPAR-α is a nuclear transcription factor found primarily in oxidative tissues such as skeletal muscle, liver, and adipose tissue [88] and upregulates β-oxidation at the transcriptional level [89]. PPAR-α upregulates the production of transport enzymes, acyl-coenzyme A oxidase and carnitine palmitoyl transferase I, which facilitate translocation of fatty acids into peroxisomes and mitochondria, respectively. Unlike, PPAR-α, PPAR-γ is expressed primarily in adipocytes, and it facilitates efficient storage of lipids by upregulating the expression of lipoprotein lipase, fatty acid transport protein, and CD36, all of which promote FFA flux into the adipocyte and triglyceride assembly [90]. As discussed, efficient lipid storage is important in the prevention of insulin resistance, as excess lipids within circulation can deposit in muscle tissue [51]. The antidiabetic drug class of thiazolidinediones are a PPAR-γ agonist and improve insulin resistance in nondiabetic obese subjects [91]. In an animal model, PPAR-γ knockout mice exhibit increased serum FFA and insulin resistance [92]. Dietary ligands for this family of PPARs include MUFA, which is a much more sensitive ligand than SFA [89]. Thus, nuts, which are rich in MUFA, may upregulate lipid metabolism and improve insulin sensitivity by upregulating PPAR-α and PPAR-γ, potentially leading to decreased body weight [93]. However, this pathway mediated by nuts has not been directly experimentally tested.

### 2.6. The Role of Polyphenols on Uncoupling Proteins (UCP) and PPAR

Plant-based foods are a rich source of phytochemicals, which can serve as ligands, substrates, inhibitors, and cofactors for a variety of enzymes [94]. The consumption of phytochemicals, particularly polyphenols, which are present in a variety of plant foods (e.g., berries, grapes, onions, apples, cacao, green tea, soy, whole grains, etc.), are associated with reduced mortality and chronic disease risk [95,96,97,98]. Polyphenols are hydroxylated bioactive compounds that may also impact body fat, as an inverse association between polyphenol consumption and body weight has been observed [99,100]. In fact, in a randomized, interventional trial consisting of 17 obese, middle-aged men and women, 12-week consumption of 370 mg/d of polyphenols extracted from grapefruit, green tea, grape, black carrot, and guarana seed resulted in a 6.7% reduction in body mass and 7.1% reduction in fat mass in obese subjects compared to placebo [101]. Further, in a three-arm, randomized trial, obese subjects with metabolic syndrome consumed 4 cups/d of green tea (*n =* 13) or the phenolic equivalent of a green tea extract (~900 mg catechins; *n =* 10) for 8 weeks. In both green tea and green tea extract groups, significant reductions in body weight were observed (−2.5 kg and −1.9 kg, respectively) compared to control (*n =* 12) [102].

The upregulation of mitochondrial membrane uncoupling proteins (UCP) may be partly responsible for these effects. While mitochondrial oxidative phosphorylation is coupled to ATP synthesis, basal proton (H^+^) leak through the inner mitochondrial membrane generates heat [103], a significant contributor to basal thermogenesis. Similarly, UCP also captures free H^+^ to generate heat and is involved in cold-induced thermogenesis [104]. Several UCP isoforms exist: UCP-1 comprises up to 10% of total mitochondrial protein in brown and beige adipose tissue, while UCP-2 and UCP-3 are found in much smaller quantities, 0.01% and 0.1%, respectively [104]. While both unsaturated and SFA can upregulate UCP expression [105,106], polyphenols may also target UCPs. In 3t3-L1 adipocytes, the green tea polyphenol, (−)-epigallocatechin-3-gallate, in a dose-dependent manner (0–10 μM), promoted an increase in UCP-2 mRNA over the course of 24 h [107]. Tea catechins in vivo also resulted in increased UCP-1 expression in brown adipose tissue and decreased white adipose tissue mass in male rats fed a high-fat 500 mg TC/100 g chow diet for 8 weeks [108]. In addition, resveratrol consumption (4 g/kg of food) in mice increased UCP-1 expression in brown adipose tissue [109].

Besides upregulating thermogenesis resulting from increased UCP expression, polyphenols can also target PPARs [110], thus improving insulin sensitivity and potentiating the effects of thermogenesis. For example, 12-week consumption of yerba mate, a rich source of polyphenols, at a dose of 1g/kg by mice fed a high-fat diet reduced body weight, epididymal fat mass, and increased PPAR-γ expression [111]. Similar effects on PPAR-γ were observed in mice fed a high-fat diet supplemented with 1% sorghum powder, another rich source of polyphenols, for 14 weeks [112]. While body weight was nonsignificantly lower in mice consuming a 1% sorghum high-fat diet compared to control high-fat diet (38.44 vs. 41.44 g, respectively), PPAR-γ was significantly increased, which coincided with significantly decreased fasting glucose (7.14 vs. 10.01 mmol/L) and insulin (59.53 vs. 120.58 pmol/L) indicating increased insulin sensitivity. Additional animal studies have also indicated upregulation of β-oxidation in liver, muscle, and adipose tissue via increased PPAR-α expression [113,114,115,116,117]. Thus, polyphenols can act in a multitargeted approach to increase thermogenesis and reduce body weight.

Although olive oil is one of the most calorically dense foods (Figure 2), it also contains both polyphenols [118] as well as unsaturated fatty acids, which theoretically should contribute to reduced body weight. A 3-year follow-up of the Prevención con Dieta Mediterránea (PREDIMED) study, in which subjects (*n =* 7447) were randomly assigned to either a control diet or to consume nuts (5 g/d walnuts, 7.5 g/d hazelnuts, and 7.5 g/d almonds) or olive oil (50 g/d), energy density was not associated with increased body weight [119]. In a randomized trial, overweight women consumed either 25 mL of soybean oil (*n =* 20) or extra virgin olive oil (*n =* 21) for breakfast as part of a hypocaloric diet [120]. Insulin sensitivity was improved to a greater extent in those consuming extra virgin olive oil (*p =* 0.054), and body fat was reduced to a greater extent in the olive oil group compared to the soybean oil group (*p =* 0.072).

In contrast, 12 overweight, diabetic subjects were assigned to consume a higher carbohydrate diet plus ~1 tbsp of olive oil/d or a high monounsaturated fat-rich diet including ~4 tbsp of olive/d with similar total caloric intakes (~1950 kcal/d) for 6 weeks, but no significant changes were observed in body weight between the two groups [121]. Further, in a 3-week randomized, crossover study, middle-aged and overweight men and women consumed either 4 tbsp of corn oil (*n =* 27) or extra virgin olive oil (*n =* 27) as part of a ~2400 calorie diet [122]. Body weight did not change following the intervention in either group. However, based on the evidence, to claim that olive oil can be used as a weight-loss strategy would be misleading. In fact, a very-high olive oil diet could potentially elicit weight gain despite energy compensation from thermogenesis. For example, rats consumed a 25% extra virgin olive oil diet for 20 weeks, after which they became obese and insulin resistant [123]. Thus, healthy subjects consuming olive oil in moderate quantities may not elicit weight gain due to increased UCP [124,125] and PPAR expression [89]. However, feeding studies examining the effects of olive oil on body weight in which olive oil is added on top of basal calories have not been conducted. Thus, adding olive oil to one’s diet cannot be recommended for overweight or obese subjects as part of a weight-loss strategy, as it is unclear as to whether increased UCP and PPAR would compensate for the high caloric density of the food when daily calories are in excess from adding olive oil.

## 3. Considerations for Health beyond Weight Loss

### 3.1. Plant-Based Versus Animal-Based Diets for Weight Loss

Those consuming plant-based diets typically have a much higher percentage of calories derived from carbohydrates (~10% median increase in percent carbohydrates in vegans vs. omnivores) [126]. Despite the consistent ability of plant-based diets to reduce body weight, paradoxically, diets very high in animal-based foods and low in carbohydrates may also reduce body weight [127]. Low-carbohydrate diets are typically higher in SFA, and whole grains, legumes, and fruits are minimized. However, reductions in body weight (a) do not necessarily translate to reductions in fat mass and (b) do not directly translate to improved health outcomes. 

A review of human metabolic trials demonstrates that, calorie for calorie, restricting dietary fat results in more weight loss than restricting dietary carbohydrates [128]. For example, in a randomized, controlled-feeding study with obese men and women (*n =* 19), estimated reductions in grams of body fat per day from restricting dietary carbohydrates was estimated to be 53 g/d; however, restricting dietary fat resulted in 89 g/d of body fat loss [129]. Interestingly, over the course of 6 d, body weight reductions were greater in those reducing dietary carbohydrates compared to those reducing dietary fat (−1.85 vs. −1.3 kg). Nonetheless, those restricting dietary fat lost more body fat than those restricting dietary carbohydrates. These results illustrate the discrepancy between reduced body weight and reduced body fat in the case of low-carbohydrate diets derived primarily from animal-based foods. Initial losses in body weight on such diets may be due to loss of lean muscle, both from glycogen depletion and break down of skeletal muscle to release amino acids for gluconeogenesis. Thus, restricting dietary carbohydrates, which are the bulk of plant-based foods that are beneficial to health (e.g., whole grains [130], legumes [131,132], and fruits [133]), may not be a desirable strategy for weight loss.

In a 4-week, repeated measures metabolic ward study evaluating the effects of a ketogenic diet (15% protein, 5% carbohydrate, 80% fat) compared to a baseline diet (15% protein, 50% carbohydrate, 35% fat) in 17 men (age 18–50 years, BMI 25–35 kg/m^2^), urinary nitrogen significantly increased, indicating increased protein utilization, which coincided with decreased fat loss over the course of the intervention [134]. Unsurprisingly, fasting serum FFA during the ketogenic diet increased from 0.479 to 0.803 mmol/L, a FFA concentration typically present in those who are diabetic, obese, or insulin resistant [135]. Indeed, it has been observed in a secondary analysis of this trial that the ketogenic diet resulted in a significant degree of insulin resistance as assessed by homeostatic model assessment of insulin resistance (HOMA-IR) [136]. Furthermore, low-density lipoproteins and high-sensitivity C-reactive protein (hs-CRP) significantly increased, suggesting increased CVD risk. 

Low-carbohydrate diets may also negatively impact artery function [137,138], indicative of oxidative stress and inflammation [139]. Additionally, preliminary data suggest that an Atkins diet, characterized by very high animal protein intake, significantly reduced myocardial perfusion and increased hs-CRP and lipoprotein(a) over the course of 1 year in those with CVD [140]. A comprehensive review of prospective studies found that those consuming the least carbohydrates (<40% of energy) had a much higher mortality rate compared with median carbohydrate intake (50–55% of energy) in a dose-dependent manner [141]. While those with higher-carbohydrate intakes (>70% of energy) also had higher mortality, this effect was not apparent when carbohydrates were derived from whole plant-food (e.g., whole grains). Collectively, these data suggest that chronic disease risk may be promoted by diets derived primarily from animal-based foods. 

### 3.2. Health Effects of Plant-Based Diets

Plant-based diets are associated with reduced mortality, particularly from CVD and cancer [142]. Indeed, interventional trials have revealed that plant-based diets can reverse atherosclerosis and improve myocardial perfusion [140,143,144], an effect exclusive to plant-based diets. These effects may be attributed to decreased inflammation and oxidative stress [21,145]. Clinical evidence also suggests that plant-based diets may facilitate prostate cancer regression [146], and preliminary data indicate improved risk factors associated with breast cancer [147]. Pilot data in subjects previously diagnosed with prostate cancer suggest that a plant-based diet can increase telomere length compared to a control group receiving standard care, suggesting the potential for an extended lifespan [148]. As such, plant-based diets may provide benefits in the prevention of chronic disease beyond reduced fat mass.

It should be noted that plant-based diets derived from refined or processed sources would not be expected to elicit beneficial effects including weight loss, reduced inflammation, and mortality as discussed. For example, in a composite prospective study including individuals from the Nurses’ Health Study 1 and 2 as well as the Health Professionals Follow-up Study (*n =* 116,969), dietary patterns of unhealthy plant-based diets (uPD) versus healthy plant-based diets (hPD) were documented [149]. Foods comprising uPD were defined as fruit juice, refined grains, potatoes, desserts, and sugar-sweetened beverages, while a hPD comprised whole grains, fruits, vegetables, nuts, legumes, vegetable oils, as well as coffee and tea. Combined data from both uPD and hPD indicated an −8% reduced risk of CVD. However, when dietary patterns were discriminated, uPDs were associated with a 32% increase in risk of CVD, while hPDs were associated with a 25% reduction in risk of CVD. Further analysis of these cohorts found that hPDs were associated with reduced T2DM risk even after BMI adjustments (−44%) and that uPDs were associated with increased T2DM risk (+16%) [150]. In an analysis of NHANES III, similar observations were observed in relation to total mortality, as total plant-based diets and uPDs were not associated with reduced mortality [151]. However, those with the greatest adherence to hPDs had reduced mortality. Lastly, in a smaller, cross-sectional study consisting of 240 middle-aged women, hPD was significantly associated with reduced inflammatory biomarkers compared to uPD [152]. Thus, based on these data, consumption of a plant-based diet comprising unrefined, whole plant-foods can confer beneficial health effects

## 4. Conclusions

Plant-based diets can reduce body fat via a variety of mechanisms, which cumulatively lead to reduced calorie intake and increased energy expenditure. These mechanisms include reduced caloric density of the overall diet and improved satiety, in part due to increased production of SCFAs by the gut microbiota. Additionally, increased insulin sensitivity, PPAR and UCP expression, and a potential increase in beiging of white adipose tissue contribute to increased thermogenesis. Future investigations utilizing plant-based diets in the context of controlled feeding studies are warranted to establish these metabolic compensatory mechanisms. Additionally, the aforementioned proposed mechanisms require further human trials to establish the mechanistic link between plant-based diets and body fat loss.

## Figures and Tables

**Table 1:** Saturated fat content of plant- and animal-based foods.

Food Type	Total Saturated Fat g/100 g	Total Unsaturated Fat g/100g ^1^
**Animal-derived foods**		
Butter, unsalted	50.5	26.4
Cheese, cheddar	19.4	9.8
Pork, cured, bacon, baked	14.2	23.8
Cream, fluid, light (coffee cream)	10.2	5.3
Beef, ground, 80% lean, baked	6.2	9.3
Eggs, hard-boiled	3.3	5.4
Fish, salmon, Atlantic, farmed, cooked, dry heat	2.4	8.6
Milk, whole	1.9	1.0
Yogurt, Greek, plain, low-fat	1.2	0.6
Chicken breast, skin removed, baked	1.0	2.0
**High-fat plant-derived foods**		
Oil, Coconut	82.5	8
Oil, palm	49.3	46.3
Oil, Olive	13.8	83.5
Nuts, almonds	3.8	43.8
Avocados, raw	2.1	11.8

^1^ includes both monounsaturated and polyunsaturated fats. Data derived from USDA food database [56].
